# Implementation and impact of NHS-funded tobacco dependence services in England: a mixed-method evaluation protocol

**DOI:** 10.1136/bmjopen-2024-089630

**Published:** 2024-12-26

**Authors:** Maria Raisa Jessica Aquino, Kerry Brennan-Tovey, Mackenzie Fong, Angela Wearn, Theophile Bigirumurame, Tomos Robinson, Miranda Trevor, Joanna Feeney, Ailsa Rutter, Ruth Sharrock, Jane West, Sally Bridges, Angela S Attwood, Kate Jolly, Sarah Damery, Sarah Flanagan, Chris Armitage, Samantha Russell, Steve Strong, Sheena E Ramsay, Eileen F S Kaner

**Affiliations:** 1 Population Health Sciences Institute, Faculty of Medical Sciences, Newcastle University, Newcastle upon Tyne, UK; 2 Action on Smoking and Health, London, London, UK; 3 Fresh and Balance, Durham, UK; 4 Gateshead Health NHS Foundation Trust, Gateshead, Gateshead, UK; 5 Bradford Institute for Health Research, Bradford Teaching Hospitals NHS Foundation Trust, Bradford, UK; 6 School of Experimental Psychology, University of Bristol, Bristol, UK; 7 Institute of Applied Health Research, University of Birmingham, Birmingham, UK; 8 Manchester Centre for Health Psychology, University of Manchester, Manchester, UK; 9 N/A, N/A, UK

**Keywords:** PUBLIC HEALTH, PREVENTIVE MEDICINE, Implementation Science, Smoking Reduction, Health policy, Health Services

## Abstract

**Abstract:**

**Introduction:**

Tobacco smoking remains a leading cause of ill-health, premature mortality and a driver of health inequalities. To support smokers in England, a comprehensive approach to treating tobacco dependence is being implemented. This includes offering support to all people admitted to hospitals, as well as women and pregnant people within NHS settings. We aim to describe the protocol for an evaluation of this tobacco-dependence service.

**Methods and analysis:**

This is a national evaluation across five regions in England (i.e., South West, West Midlands, Greater Manchester, North East and North Cumbria, Yorkshire and Humber) including 11 NHS Foundation Trusts. It is funded from September 2021 to September 2025. Evaluation settings are acute hospital, maternity and inpatient mental health.

Work package (WP) 1 involves qualitative key informant interviews to characterise the policy implementation context. WP 2 involves an online survey to assess the routinisation of the service in practice and staff attitudes regarding tobacco dependence, plus in-depth interviews with relevant practitioners to explore survey findings and interviews with smokers to investigate its usefulness and impact. WP 3 involves descriptive statistical analysis of routinely collected data to assess service uptake and impact on health and care outcomes (e.g., smoking status). WP 4 involves an economic analysis of routinely collected data to determine the financial impact of the service. Qualitative data (WP 1, WP 2) will be analysed using Thematic Analysis and Framework Analysis, respectively. WP 2 survey data will be analysed using descriptive statistics.

**Ethics and dissemination:**

This evaluation received favourable ethical opinion from Newcastle University (17756/2021) and NHS Wales Research Ethics Committee (22/WA/0203). It has also received Confidentiality Advisory Group support (22CAG0103).

**STRENGTHS AND LIMITATIONS OF THIS STUDY**

- The evaluation uses robust mixed methods informed by theory, specifically Normalisation Process Theory and the Theoretical Framework of Acceptability.
- The evaluation is also strengthened by integrating Public Involvement and Community Engagement across all stages of the evaluation, to ensure that it is designed and delivered using approaches that are acceptable to patients and the public.
- Participants in the qualitative elements of the evaluation are self-selecting; however, a purposive sampling technique will allow for views from a diverse group of people to be captured.
- Quantitative data collection is restricted to NHS acute hospital, maternity and mental health inpatient settings (i.e., excludes data collected and reported within community-based tobacco dependence services) and could potentially limit our analyses of outcomes.
- A triangulation protocol will allow for the synthesis of data from different sources and inform recommendations for sustainable evaluation and tailoring of the service to different contexts.

## Introduction

 Tobacco smoking remains a leading cause of ill-health, premature mortality and a driver of health inequalities. An analysis of the Global Burden of Disease Study in 2019 showed that although smoking prevalence has reduced, this has not kept pace with population growth, and so the total number of smokers has increased.^1^ Moreover, progress made from tobacco control measures has slowed in the last 5 years, concluding that stronger, more consistent measures need to be implemented to achieve the Sustainable Development Goals.^1 2^ Smoking prevalence remains higher in those from lower socioeconomic backgrounds, who are also disproportionally impacted by health inequalities.^3^

The NHS Long Term Plan, published in 2017,^4^ set out health and care priorities in England over the next 10 years. This policy has a focus on prevention and health inequalities, specifically a commitment to NHS action to prevent ill-health. One key area of action was smoking, where a comprehensive package of care and treatment was implemented to provide proactive smoking cessation support to all those who were admitted to the hospital, expectant mothers and their partners and long-term users of specialist mental health services. Prior to this, the default model for tobacco dependence treatment was an opt-in approach where smokers could seek help to quit from healthcare professionals, with the exception of maternity services where opt-out pathways have been recommended since 2010.^5^ This paper describes the protocol for a mixed-method evaluation of this tobacco-dependence treatment service in acute hospitals, maternity and inpatient mental health settings in five regions in England, which aims to assess its implementation and impact on health and care outcomes.

### NHS-funded tobacco dependence services

The NHS-funded tobacco dependence services (NHS TDS) is the package of care and treatment for people who smoke in England. This includes three bespoke models for inpatient hospital, maternity and inpatient mental health settings, which include a combination of screening, treatment (e.g., freely available nicotine replacement therapy (NRT)) and behavioural support, as summarised in table 1. The aim of this study is to undertake an evaluation of the implementation of the NHS TDS.

**Table 1:** NHS-funded tobacco dependence services (NHS TDS) care models by setting

Setting	Care model
Inpatient acute	A patient’s smoking status is recorded at admission. Patients are provided very brief advice and a referral for a consultation with an in-house service. They are then provided a personalised treatment plan which includes: behavioural support, nicotine replacement therapy (NRT) or other forms of pharmacotherapy during their hospital stay, with follow-up at 28 days post-discharge. Patients are then discharged into community services where appropriate.
Maternity	Adapts the inpatient acute model (above), delivered in-house within maternity services. In addition, all women will be offered a carbon monoxide (CO) test at the first antenatal booking appointment, with maternity staff recording the outcome, followed by a one-on-one meeting with a tobacco dependency advisor (TDA) arranged at the first antenatal booking appointment.Weekly face-to-face appointments with the TDA take place for at least 4 weeks, with NRT supplied for up to 12 weeks beyond the quit date. Support is continued throughout the pregnancy with appointments at least monthly. A 28-day quit status is recorded by the TDA, followed by a CO-validated smoking status at 36 weeks and an updated smoking status is recorded at time of delivery.
Inpatient mental health	Adapts the inpatient acute model (above), delivered in inpatient mental health settings. While all TDA should aim to see all patients within 48 hours of admission, it is understood that this may not be possible due to the nature of mental illnesses; likewise, the initial TDA assessment may not be complete in one session and may require multiple visits.Additional weekly face-to-face appointments with TDA are expected, to ensure engagement and monitor progress for the first 4 weeks of admission. Prior to discharge a face-to-face planning session between the patient and TDA to identify where support and medication can be accessed post-discharge; this support continues beyond discharge in line with mental illness and/or local pathways.

These models are adapted from evidence-based smoking cessation interventions; in particular, the Ottawa Model for Smoking Cessation (OMSC, Canada) and the Conversation, Understand, Replace, Experts and Evidence-Based treatment (CURE) project within the UK. The OMSC uses a ‘5 As’ approach—ask, advise, assess, assist and arrange.^6^ This model aims to support smokers in achieving long-term smoking abstinence after being admitted to the hospital through documenting inpatients’ smoking status; providing brief advice and pharmacotherapy; opt-out referral to specialist teams for inpatient and post-discharge behavioural support via community-based services.^7^ The CURE project is the closest adaptation to the OMSC model in England, implemented in acute hospital settings. CURE adopted a whole-system approach in line with the National Institute for Health and Care Excellence (NICE) PH48 guidelines (section 4.1). On admission to the hospital, patients are screened for smoking status. On identification and recording as an ‘active smoker’, the individual is referred to the CURE team, provided brief advice and offered NRT and varenicline at no cost to the smoker. Within 48 hours of admission, further support is provided including a 45-min consultation with a specialist stop smoking practitioner, a review of NRT and additional medications, behavioural support, signposting to post-discharge support and a follow-up plan developed. After discharge, a telephone follow-up is conducted between weeks 1 and 2; then a face-to-face 4-week follow-up with CO verification of smoking status; and finally, a 10–12-week telephone follow-up. The model is tailored for maternity settings, such that continued support is provided throughout pregnancy with smoking status recorded at delivery; and for mental health, the frequency of assessments and follow-up support can increase due to the nature of mental illnesses.

### Evaluation aims and objectives

The specific objectives for each study component are summarised in table 2. Implementation of the service (i.e., when the service was set up) nationally was set up to begin between in 2021/2022. Trusts participating in this evaluation started service implementation between February 2022 and November 2022, following challenges to service and data collection setup across the country. These studies are complementary to the national evaluation undertaken by NHS England (https://digital.nhs.uk/data-and-information/publications/statistical/statistics-on-nhs-stop-smoking-services-in-england).

**Table 2:** Evaluation objectives and study components

Evaluation objective	Study components
To describe the implementation context, including practices, services and structures of pre-policy implementation	Work package (WP) 1: Key informant interviews and document analysis
To explore system-wide implementers’ (ie, front-line staff, managers or service leads, service commissioners) perceptions and experiences of service implementation	WP 2a: Survey of healthcare professionalsWP 2b: Interviews with healthcare professionals
To explore smokers’ perceptions of service implementation	WP 2c: Interviews with smokers offered the NHS-funded tobacco dependence services (NHS TDS)
To determine the level of participation in the NHS TDS	WP 3: Analysis of routinely collected patient data (effectiveness and process analysis)
To estimate service effectiveness as the change in number of smokers who have undertaken an intervention and self-reported and/or have a carbon monoxide (CO) confirmed 28-day quit	WP 3: Analysis of routinely collected patient data (effectiveness and process analysis)
To estimate the cost-effectiveness of the service per additional quit	WP 4: Health economic analysis

### Evaluation sites

This evaluation will take place in five regions across England: West (Bristol), North East and North Cumbria, West Midlands, Yorkshire and Humber and Greater Manchester. As of 2021, the average smoking prevalence among adults in England aged 18 and over is 13%.^8^ All regions in the evaluation have smoking rates higher than those of the national average: Bristol at 16%, the North East at 15%, West Midlands at 14%, Yorkshire and Humber at 14% and Manchester at 17%.^8^ As of 2022, the average smoking prevalence at the time of delivery in England was 8.8%. Four regions had higher smoking prevalence than the national average: North East at 12.5%, West Midlands at 11.4%, Yorkshire and Humber at 11.6% and Manchester at 9.2%. However, Bristol was slightly lower at 8.4%. Regarding mental health, as of 2022, smoking prevalence among adults with a long-term mental health condition in England was 25.2%.^8^ Four regions were higher than the national average: West Midlands at 25.5%, Manchester at 31.0%, Bristol at 30.4% and Yorkshire and Humber at 27.5%; however, North East of England was lower with 23.5%.^8^

## Methods and analysis

A mixed-method evaluation of effectiveness and implementation will be undertaken to explore the impact of the service on health and care outcomes.^9^ This hybrid approach will integrate analysis of qualitative interviews, document analysis and surveys with analysis of routinely collected patient data and health economic data.^9^

We developed an initial logic model based on the NHS-funded tobacco dependence implementation plan across the three settings (table 1), and additional documentation provided by the Office for Health Improvement and Disparities (e.g., detailed treatment pathways for each setting, via email), illustrated in table 3 below. This representation of pathways from implementation strategies and mechanisms to an intervention’s outcomes will be amended using evidence generated from work package (WP) 1, key informant interviews and document analysis. The logic model presents the anticipated outcomes of implementing this intervention in the short, medium, and long terms; however, not all outcomes will be assessed as part of this evaluation. Details on outcomes relevant to this evaluation are reported in their respective WPs (where applicable).

**Table 3:** Initial logic model for the implementation of the NHS-funded tobacco dependence services

Inputs	Processes	Outputs	Outcomes and impacts		
			Short term	Medium term	Long term
Funding to deliver the tobacco dependency service (TDS) including the provision for further staff Staff needed to deliver the intervention within the TDS The time needed to understand the process changes (staff training, technology changes, etc) Technology (record keeping, medication dispensing, referrals, communication with General Practitioners (GPs) and local stop smoking services (SSS)) Long Term Plan (LTP), National Policy and Trust Policy Regional TDS protocol/implementation plans Trust protocol development Communication pathways between the admitting team, TDS team, GPs and SSS Creation of training packages	Staff training in very brief advice (VBA) and nicotine replacement therapy (NRT) Recruitment of additional staff Creation of a TDS team of specialised staff (and its fitting in the wider hospital teams) Stakeholders and staff involvement in protocol/trust policy development and delivery Implementation and enforcement of protocols Technology adapted to record new data collected regarding smoking status Medication protocols for prescribing (NRT) and tobacco dependency (TD) medications Space for recording NRT preferences and prescription Kardex (or online system, that is, ePMA) Documentation of TD medication/NRT administration Creation of a/multiple referral scheme(s)/pathway for admitting staff/TDS staff/community support post-discharge Communication channels between staff/TDS/community/GP	Patients screened for smoking status in acute/maternity/mental health Delivery of intervention (dependent on pathway) VBA provided and NRT prescribed to those identified as smokers Tobacco dependent advisor (TDA) to provide a comprehensive assessment of identified smokers needs TDA to provide an assessment, CO testing, NRT options and behavioural advice Discharge with ongoing support or onwards referral in the community for inpatient pathway/and adapted for MH pathway. Maternity will provide ongoing support Provide follow-up as protocol dictates	Increase in smoking status recording Increase in NRT prescribing Increase in quit date set Increase in CO recording (will be dependent on if recording systems allow for that in inpatient, currently data metrics from NHS do not have requirement for this)	Continuation of support in the community Reduced length of hospital stay Increase the number of quitters being supported in the community & referrals to community services on discharge Better clinical management of illness in hospital. Reduction in re-admission Bed days saved Reduction in smoking on hospital sites Reduction in NHS staff smoking Increase in partners attempting to quit during pregnancy More smoke-free homes during pregnancy Hospital sites being smoke free	Long term quit (with/out community support) Reduced smoking-related admissions Reduction in smoking-related illness and deaths Reduction in years of disability Increase in quality of years of life Reduction in smoking population Reduction in NHS staff smoking Change in staff attitude towards smoking, not a lifestyle choice but medical condition
**Unintended consequences/dark logic model**^33^ Risk of widening inequalities and/or barriers to participation for marginalised groups. Risk of increased tension between primary and secondary care. Increased time pressures and paperwork for staff. Potential pressure on Local Authority Stop Smoking Services pharmacotherapy budgets.					

### Programme setup—pre-evaluation activities (months 1–12)

The first year of funding will be used to appoint researchers across the evaluation regions, obtain the relevant ethical approvals and data-sharing agreements and complete procedures for setting up participating NHS Trusts.

### WP 1: key informant interviews and document analysis (months 13–24)

#### Design

This qualitative WP aims to characterise the implementation context, using key informant interviews and document analysis. It will describe currently available smoking cessation services, provisions and structures in each of the evaluation regions, as well as NHS TDS implementation plans. Data will facilitate the refinement of the logic model described in the preceding section.

#### Sampling and recruitment

Participants will be purposively selected from all three settings (table 1) based on their relevant knowledge and role to ensure that the most relevant informants are interviewed. This will include front-line healthcare professionals (e.g., doctors, nurses, specialist smoking cessation staff) from all three settings, and service commissioners and managers responsible for tailoring/leading the implementation of the service. Participants will be recruited through key contacts in each of the geographical regions (via email) combined with a snowballing approach.

### Data collection

#### Interviews

We will conduct semi-structured interviews across the five evaluation regions. Interviews will be conducted by members of the research team local to each region, either face-to-face (in line with COVID-19 restrictions), telephone or secure videoconferencing platform (e.g., Teams/Zoom). Interviewers will be experienced researchers, from related disciplinary backgrounds (eg, public health, behavioural science), with no prior relationship with or knowledge of participants and their backgrounds. A bespoke topic guide (online supplemental file 1) informed by existing literature and discussions with the research team^10^,^13^ has been pilot-tested and will be used to guide conversation. All interviews will be recorded and transcribed verbatim. Transcribed data will be anonymised, with participants’ names replaced with unique participant IDs. Digital recordings and digital transcripts will be stored on a secure server. Data collection and recruitment will be guided by data saturation and information power, as aligned with the evaluation aims and proposed methods (including application of theory and analytical method).^14 15^

#### Document analysis

We will source relevant documents including, but not limited to hospital policies, implementation plans, and protocols concerning the NHS TDS from the five evaluation regions (eg, through Smoke Free NHS Leads, Treating Tobacco Dependency Taskforces within Integrated Care Systems). National-level policy is publicly available online.

#### Analysis

Interview and documentary data will be managed using NVivo software^16^ and will be analysed thematically using established methods by members of the research team.^17^ Interview data analysis will be inductive, that is, data-driven, following a six-phase iterative process of coding data and theme generation. Documentary data will be coded using the Template for Intervention Description and Replication (TIDieR) framework, comprised of 12 items that will allow us to detail the rationale and procedures for intervention delivery and implementation settings.^18^ Data analysis will follow a six-phase approach in line with Braun and Clarke’s Thematic Analysis.^17^

### WP 2: surveys and interviews with healthcare professionals and interviews with smoking individuals (months 25–48)

#### Design

This mixed-method WP aims to explore staff and smoker perceptions of NHS TDS implementation and receipt, respectively, using a longitudinal quantitative survey and semi-structured interviews. Specifically, it aims to investigate barriers and enablers to NHS TDS delivery, explore how staff attitudes regarding smoking changes over time and explore smokers’ experiences of being offered, or receiving, NHS TDS.

### Sampling and recruitment

#### Healthcare professionals

Healthcare professionals involved in the implementation of NHS TDS in acute hospital, maternity or mental health settings in any of the evaluation regions will be eligible to take part. We define involvement as healthcare professionals as those who are expected to or have started to deliver NHS TDS in the above-mentioned settings (table 1). We will use a combination of convenience and snowball sampling techniques to recruit participants.

#### Smokers

Adults (aged 18 and over) who are identified as smokers in acute hospital, maternity or mental health settings and offered the service in any of the evaluation regions will be eligible to participate in interviews. Eligible participants will be identified by a healthcare professional and will seek consent from them to be contacted by the research team. We aim to interview a range of individuals across implementation settings and evaluation sites, for example, those who have been offered and engaged with the service; offered and declined the service and offered and engaged with the service then subsequently withdrawn. Participants will also be purposively selected based on demographic characteristics such as gender and ethnicity to capture a diverse range of perspectives on the service.

### Data collection

#### WP 2a—survey

An online survey using two validated tools—using the NOrmalization MeAsure Development (NoMAD) questionnaire,^18^ and the Capabilities, Opportunities, Motivations and Behaviour Model (COM-B) questionnaire^19^—will be sent via email to healthcare professionals at two time points. The first survey will be circulated as close as possible to the beginning of the implementation period, with the second survey circulated 6 months after the first. The NoMAD questionnaire is useful for understanding how interventions are routinised into practice, and the COM-B questionnaire is useful for understanding barriers and enablers to enacting behaviours (i.e., delivering NHS TDS). Participation is voluntary and participants will be recruited by email through their service managers/leads. Invitation emails will include a Participant Information Sheet and a link to participate in the survey. Within the survey, there is the opportunity to detail reasons for non-participation, should they choose to decline.

#### WP 2b—interviews with healthcare professionals

Interviews with healthcare professionals approximately 12 months after intervention implementation. We anticipate 30 one-off semi-structured interviews either face-to-face, phone or secure videoconferencing platform (e.g., MSTeams/Zoom). A bespoke topic guide (online supplemental file 2) informed by NPT^20 21^ will be used to explore healthcare professionals’ experiences of implementing the NHS TDS. Interviews will last no longer than 1 hour and will be audio-recorded or video-recorded and transcribed verbatim by a professional transcription company.

#### WP 2c—interviews with smokers

We anticipate approximately 50 one-off semi-structured interviews with smokers (based on the sampling criteria above) either face-to-face, phone or secure videoconferencing software (e.g., MS Teams/Zoom). A bespoke topic guide (online supplemental file 3) informed by TFA^22^ will be used to explore smokers’ experiences of the tobacco dependence service. Interviews will last approximately 1 hour and will be audio-recorded or video-recorded and transcribed verbatim by a professional transcription company. Data collection and recruitment will be guided by data saturation and information power, as aligned with the evaluation aims and proposed methods (including the application of theory and analytical method).^14 15^

#### Analysis: survey

Data will be managed using STATA.^23^ Questionnaires will be analysed descriptively, using absolute (n) and relative frequencies, means and SD as appropriate to the level of data, and 95% CI.^24^ Mean scores for each of the four NPT constructs and the six COM-B constructs will be calculated.^25^ Higher positive scores on NPT constructs are suggestive of greater potential for the NHS TDS to ‘normalise’ or become routinised.

#### Analysis: interviews

Interview data will be managed using NVivo^16^ and will be analysed using framework analysis^26^ by members of the research team. The framework method is systematic in allowing us to use coding frameworks informed by either NPT or TFA. While offering the flexibility of being able to derive themes inductively to capture implementation issues that might not fit ‘neatly’ into either NPT or TFA constructs.

### WP 3: effectiveness and process analysis (months 25–48)

#### Design

This quantitative non-randomised observational study aims to assess the reach of the service as well as the effect on health outcomes (effectiveness) and healthcare use (service uptake).

#### Population

All adults (aged 18 and over) who are identified as smokers in acute hospital, maternity or mental health settings, are offered the service in any of the participating NHS Trusts within the five evaluation regions and have recorded notes available to the research team between December 2022 and March 2025 will be included in this analysis of individual patient data. While no formal sample size calculation was conducted, we estimate approximately 69 000 potential individuals for inclusion in the evaluation. This estimate was based on 11 participating NHS Trusts, approximately 7 months of data collection (taking into consideration potential data governance and submission delays/gaps), and a national smoking prevalence of 12.1% in England.

#### Data collection

Data will be collected across acute, maternity and mental health settings. These will include individual patient-level data drawn from medical records. The data will be used to determine:

Process outcomes: The service delivery and uptake (ie, the number of inpatients with smoking status recorded), the number of patients referred to the in-house service, number of those who were referred and were seen, the number of those who were referred who do receive treatment while in hospital, and the number of those who were referred to additional services on discharge.

Behaviour/health outcomes: Post-discharge smoking status (28-day smoking status, carbon monoxide-validated smoking status, smoke-free births). In acute inpatient and mental health inpatient will include either 28-day smoking status or self-reported quit. In maternity settings, quit data will include carbon monoxide-validated smoking status, smoking status at 36 weeks, smoke-free births, or self-reported quit.

#### Analysis

Findings will be reported descriptively. Frequencies and percentages (with 95% CI) will be reported. Subgroup analysis will be conducted and involve age, ethnicity, gender, regions, smoking status at admission, referral to in-house tobacco treatment team, tobacco care plan, onwards referral made, 28-day smoking status and smoking status at the time of delivery and at 36 weeks. We will also stratify findings by Lower Super Output Areas and Index of Multiple Deprivation, to explore whether there are inequalities in service delivery/uptake.

### WP 4: health economic analysis (months 25–48)

#### Design

This quantitative non-randomised observational WP aims to explore the cost of offering the service and if sufficient high-quality data are available to estimate the return on investment in the form of a cost per quit.

The economic evaluation will be undertaken from the perspective of the healthcare provider. A costing analysis will be conducted to explore the cost of providing the service once it has been implemented in a trust.

#### Population

Adults (aged 18 and over) who are identified as smokers in acute hospital, maternity or mental health settings and offered the service in any of the participating NHS Trusts within the five evaluation regions. While no formal sample size calculation was conducted, we estimate approximately 69 000 potential individuals for inclusion in the evaluation (based on 11 participating NHS Trusts, approximately 7 months of data collection, and a national smoking prevalence of 12.1% in England).

#### Data collection

Data on quit rates will be collected across acute, maternity and mental health settings. Quit data for acute inpatient and mental health inpatient will include either 28-day smoking status or self-reported quit. In maternity settings, quit data will include carbon monoxide-validated smoking status, smoking status at 36 weeks, smoke-free births or self-reported quit. All individual patient-level data will be drawn from medical records. Data on costs will be determined from findings from WP 1 and WP 2, discussions with the wider project team, appropriate information gathered from the different trusts, and previous studies in this area.^27^

#### Analysis

The health economic analysis will take a bottom-up approach to estimate the cost of providing the service per service user once it has been implemented in the trust. Where sufficient data are available, a cost per quit will be estimated.

A model of a service user’s pathway through the service will be developed based on the draft pathways provided in the protocol. Each component of the pathway will be costed in collaboration with service managers. The pathway will be altered as required to meet the trust-specific service pathway. The cost for the full pathway will be calculated per service user.

The primary analysis will be at a trust level; however, if sufficient data are available, a pathway-specific cost for the three different pathways (acute, maternity, mental health) will be calculated.

The perspective of the costs will be the healthcare provider and will be based on the standard approach used in economic evaluation, which involves identifying, measuring and valuing all appropriate costs.^28^ Several cost categories will be considered, including staff costs (eg, tobacco treatment advisors) and ongoing non-staff costs (such as consumables and training courses). Costs will be calculated in Great British pounds corresponding to prices in the ‘cost year’ of the evaluation (2021/2022). Where required, relevant unit costs will be gathered from routine data sources.^29 30^

Where sufficient data are available, the costs of the intervention will be combined with the quit rates calculated in WP 3 to estimate a return on investment (in the form of an estimated cost per quit). Return on investment can be thought of as representing the ‘rate of return’ from an investment.^31^ If sufficient data are available regarding the different pathways (acute, maternity, mental health) a separate estimated cost per quit will be calculated for each pathway.

### Integration and synthesis of all WPs

**Figure 1:**
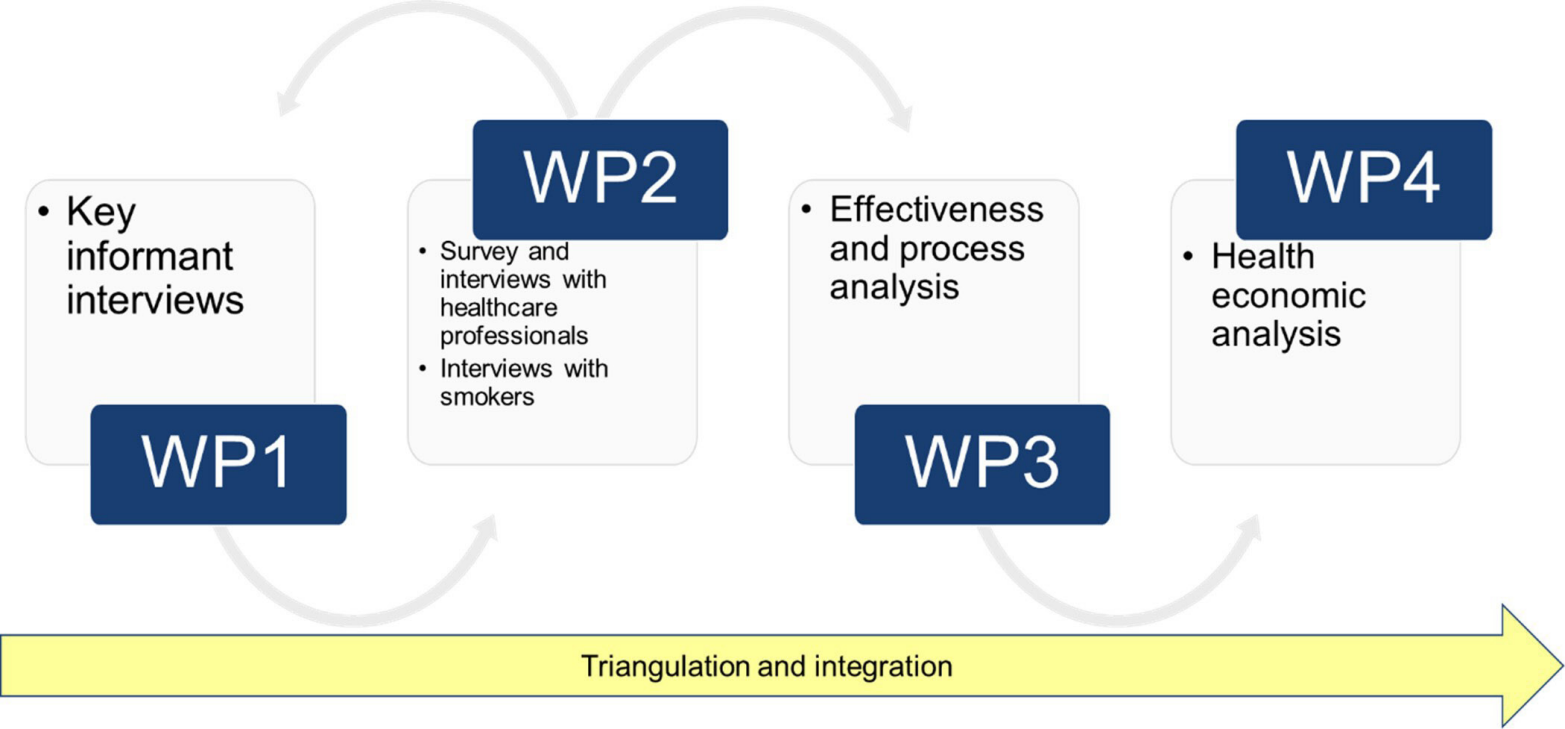
Evaluation flowchart (in work packages).

To triangulate and integrate the data from the four work packages, we will apply methods suggested by Farmer *et al*.^32^ Integration will involve identifying key themes or findings from each of the work packages and grouping these. Then, these findings will be compared against each other. Researchers involved in the analysis will also compare each other’s interpretation of the findings. The evaluation flowchart is presented in figure 1.

### Patient, public and stakeholder involvement

The views and experiences of the public and those most affected by smoking-related illness are an integral part of this project. To ensure this research is shaped by, and reflective of, these views, we will work with a project-specific Public Advisory Group (PAG) comprised of a small group of public advisors (n=4) from across England who are either past or present smokers and/or have relevant experience of NHS inpatient acute, mental health or maternity services. The PAG will be an integral part of our wider team contributing to a number of areas including, but not limited to, study materials development, wider public engagement and recruitment plans, study analysis and outcomes and dissemination. For example, the PAG have been involved in every stage of development of all work packages, as well as write-up of the protocol. A Public Involvement and Community Engagement Lead (AW) will be embedded within the core team to ensure the public voice is prioritised and incorporated into all stages of the project.

Importantly, this programme of work was prioritised for funding by the NIHR ARC Prevention Consortium’s Stakeholder and Public Advisory Groups. Draft versions of our funding application and protocol have been reviewed by members of the public, health and care commissioners, policy stakeholders and academic partners to determine the methodological robustness and acceptability of our plans and identify areas for improvement. Feedback from this prioritisation and review process has resulted in the revision of the protocol such as ensuring that we gathered evidence on how the service fits into the wider landscape at a local level (i.e., through qualitative work and ongoing relationships with regional and national implementation partners); revising the terminology throughout our work to highlight that tobacco smoking is a chronic, relapsing condition which is a leading cause of death and disability in the UK and therefore warrants appropriate treatment and support; and developing a health economics work package to determine costs and resource-use associated with delivering the service.

## Discussion

We anticipate this mixed-method evaluation of a nationally provided tobacco dependence service to generate evidence regarding its implementation, drawn from all settings (i.e., acute hospital, maternity and inpatient mental health), across five regions in England. Localised and context-dependent implementation strategies, both successful and unsuccessful, will form recommendations for future iterations of this national policy. Moreover, the evaluation will support national-level assessment of the impact of the service on health outcomes, through planned statistical and health economic analyses. Taken together, integrated data from the various work packages will allow us to identify variations in service provision, and make recommendations for enhancing implementation, tailored to local contexts, to mitigate the risk of widening health inequalities. Our ongoing and sustained relationships with lay, clinical, policy, academic and practice partners will also support the co-production of key findings from this work, which will be disseminated widely, to advance research, policy and practice relating to addressing tobacco dependence in England.

## Ethics and dissemination

Favourable ethical opinion for the evaluation was sought from the appropriate ethical committees prior to the recruitment of participants commencing at any site.

For WP 1, ethical approval was sought and obtained from Newcastle University (17756/2021). For WPs 2–4, ethical approval was sought and obtained from NHS Wales REC 5 (22/WA/0203), and Confidentiality Advisory Group (CAG) support was obtained (CAG: 22CAG0103).

Findings from this evaluation will be disseminated to all relevant stakeholders (i.e., academic, practice, policy and public partners) at regular intervals, to inform the implementation and development of the service. Policy briefs for dissemination to practice and policy partners will be co-developed with these stakeholders to ensure all relevant information is captured. We will include public partners as co-authors in all evaluation outputs (e.g, conferences, peer-reviewed articles, policy briefs/summaries, final report).

## supplementary material

10.1136/bmjopen-2024-089630 online supplemental file 1

10.1136/bmjopen-2024-089630 online supplemental file 2

10.1136/bmjopen-2024-089630 online supplemental file 3
