# Supplementary material for: Implementation and impact of NHS-funded tobacco dependence services in England: a mixed-method evaluation protocol
Source: BMJ Open. 2024 Dec 26;14(12):e089630. doi: 10.1136/bmjopen-2024-089630 (PMC11683999; doi:10.1136/bmjopen-2024-089630)
Supplement: online supplemental file 1 [file bmjopen-14-12-s001.docx]

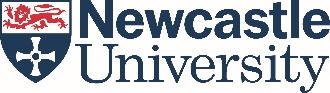

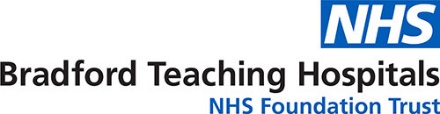

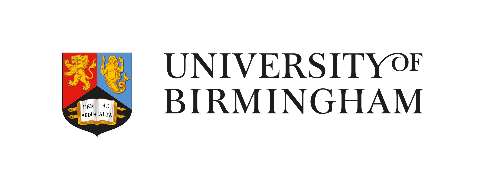

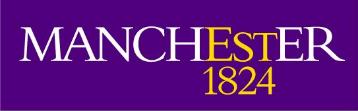

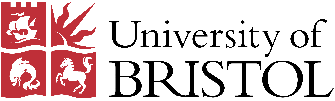


**Supporting the NHS Long Term Plan: An evaluation of the implementation and impact of NHS-funded tobacco dependence services**

**Key informant interview topic guide**

Thank you for agreeing to take part in this interview. We are inviting a number of health care professionals, service commissioners and managers across England, so I am pleased you are able to speak to us today. We are interested in finding out more about existing tobacco dependence support services in your area and your perspectives on preparing for implementing NHS-funded tobacco dependence services.

**YOUR ROLE/RESPONSIBILTIES**

• Could you give me an overview of your current occupation and responsibilities?

• How are you involved in implementing NHS-funded tobacco dependence services (and in what setting, where appropriate)?

**PRE-INTERVENTION ROLLOUT/EXISTING SMOKING CESSATION SERVICE(S)**

The following questions will help us to understand more about existing tobacco dependency services (TDS) and care pathways.

- What support is there currently, for service users in acute, mental health and maternity to manage their tobacco dependency?
  - Is there a referral pathway for post-discharge? Ask for details.
- Is patients’ smoking status currently assessed or recorded?
  - If yes, how is this done? How often is this done? And by whom?
- Are patients/service users given advice on smoking cessation?
  - If so, when is this done and in what format?
- Is smoking status of patients recorded and shared with your team?
  - Is this information shared with other teams i.e. AHP’s in order to better their treatment?
  - If so, how is this done?
- Who is there to support patients?
  - What resources are available to support patients?
- How long are patients supported by this service?
  - Are there any opportunities for follow up with patients?
- How is service impact currently measured?
  - What are the outcomes (or KPIs) of this service?
  - Are outcomes/impacts shared with staff and if so, how?
  - How have these informed your planning for the implementation of the NHS-funded TDS?
- Who is involved in delivering the existing service?
- Who is responsible for the oversight of this service?
- What are the challenges within existing service(s)?
- What are the successes of the current service model?
- How well do patients engage with this service?

**IMPLEMENTING THE LONG TERM PLAN**

We will now ask you to think about the upcoming implementation of the NHS-funded TDS as recommended by Long-Term plan.

- Could you tell me what the new pathways will/do look like in your area?
- What are the planned/applied implementation strategies for this service model?
- Who will be responsible for implementing the service (i.e. clinical leads etc.)?
- What training opportunities will be available for staff?
  - How will staff access this training?
  - What format will the training take? (i.e. face-to-face, online, shadowing etc.).
- What impact do you perceive the intervention having on the patients?
- What impact do you perceive the intervention having on the staff?
- What is your goal from the implementation of the LTP tobacco dependence service?
- What do you hope to see from the intervention?
  - In staff and patients

**BARRIERS AND ENABLERS TO IMPLEMENTING SERVICES**

- What are the [current/potential] barriers for implementing this service/model?
- What do you feel will facilitate the implementation of this service/model?
- Do you see the oversight of this service as a core part of your role?
- What motivates you to implement this service?
- How well do you believe patients will engage with the service?
- What do you think influences patients to engage with the service?
- Do you feel there may be any unintended consequences of implementing the model? These can be positive or negative.
- How will you ensure the service will be implemented and delivered as intended?
- Are there any other factors influencing the implementation of this service model that we have not already covered?
