# Supplementary material for: Implementation and impact of NHS-funded tobacco dependence services in England: a mixed-method evaluation protocol
Source: BMJ Open. 2024 Dec 26;14(12):e089630. doi: 10.1136/bmjopen-2024-089630 (PMC11683999; doi:10.1136/bmjopen-2024-089630)
Supplement: online supplemental file 2 [file bmjopen-14-12-s002.docx]

**
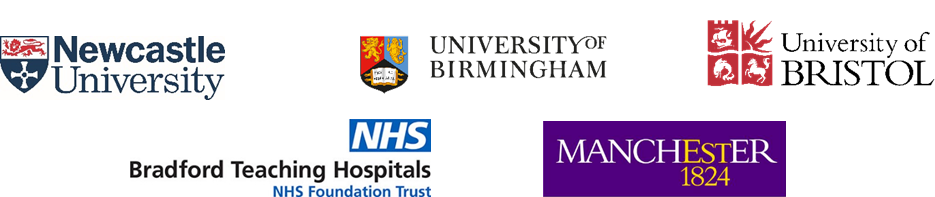
**

**Supporting the NHS Long Term Plan: An evaluation of the implementation and impact of NHS-funded tobacco dependence services**

**Healthcare providers interview topic guide**

Thank you for agreeing to take part in this interview, and for giving up your time. We are inviting a number of healthcare professions and healthcare providers across England to take part, so I am pleased that we are meeting today. We are interested in finding out more about the implementation of the tobacco dependency intervention, and explore the barriers and enablers you experienced when implementing the NHS-funded tobacco dependence service in your area.

The interview will be split into five areas, and then a chance for you to provide any further information that has not been covered, and should take no longer than 1 hour.

**YOUR ROLE AND INVOLVEMENT:**

**These first few questions are going to be around your role and involvement within the role out of the intervention:**

*Role*: Could you tell me about current occupation and responsibilities?

*Involvement*: Could you tell me what your involvement was or is within the implementation of the NHS-funded tobacco dependence services?

*Setting:* Could you provide some information about the setting that you work within, and the setting that you implemented the above intervention (i.e., acute/mental health/maternity)?

**COHERENCE (***Meanings attributed by individuals and groups to the use or utility about a practice or set of practices)*

**These next few questions are going to be around your understanding of the NHS-tobacco funded dependence service and the changes this imposed to your working role**

What is your understanding of the NHS-funded tobacco dependency service?

What is your understanding of your role within the new service?

Has this changed from your role during the rollout? (These questions may only be required for healthcare workers who have had a change in job role, as opposed as those who have been employed into the new service)

How does the NHS-funded tobacco dependency service differ from that of previous tobacco dependency services in your setting?

Did the implementation of the intervention produce any difficulties or changes in the way in which you worked? Did your role change? Examples?

How did you overcome these difficulties and changes within your work and role?

How has team working been affected when implementing the NHS-funded tobacco dependence service?

**COGNITIVE PARTICIPATION (***Engagement and commitment of people to establish and maintain collective action)*

**These next few questions are going to focus on the engagement and commitment of the people implementing the NHS-funded tobacco dependence service**

Who were the key participants in the rollout of the new tobacco dependence service? i.e. managers/commissioners

What were/are your impression of the key participants in driving the intervention forward? Examples? i.e. where they engaging? Enthusiastic?

What systems, procedures and protocols were implemented to aid the implementation of the intervention?

Did you, have any input into the systems, procedures, and protocols creation?

- If so how
- How do these systems differ to previous ones?
- Did they change the way in which you work, if so how?
- How did you find the change in these systems?

How has the whole team engaged with the intervention? Can you share some examples?

- Has team working differed/? If so, How? Examples?
- Have working relationships and dynamics changed?

What has your contribution been to the implementation?

- Has this contribution changed over the course of the implementation rollout?

How has your working changed since the intervention was implemented into your area?

What challenges does this new way of working pose? How do you overcome these challenges?

What motivators do you use to remain engaged with the intervention?

- What motivators does your setting/area use to continue to motivate staff to remain engaged with the intervention?

**COLLECTIVE ACTION** *(Actions or work people carry out or operationalise, individually or together, to achieve the goal)*

**These next questions are focusing on the actions of the team and you individually to achieve the NHS-funded tobacco dependence service**

How have you found integrating the NHS-funded tobacco dependency service into your existing work/workload?

- And work structures? Examples?

What impact do you feel the NHS-funded tobacco dependency service has had on working relationships within your team, area, and setting? Examples?

What staff training has been available to you before the intervention was implemented?

- And again, during the intervention?
- Do you feel this was adequate?
- What if anything, do you feel could make the training better?
- How often are you required to repeat or top-up the training?

Do you feel you and your team have the appropriate skills necessary to deliver the NHS-funded tobacco dependency service intervention as required?

What resources have been made available to you to effectively implement the intervention?

- your team,
- geographical area
- setting (i.e. acute; mental health; maternity services)
- Are they sufficient and adequate?

What resources do you feel need to be made available that may not have been?

How has your management team supported your role within the intervention, and the implementation as a whole? (i.e., do they lead by example?)

- Has this support allowed for effective working?

**REFLEXIVE MONITORING** *(Peoples appraisal or evaluation, formal or informal, of the practices implemented and its impact on themselves or others)*

**These questions focus on your assessment of the NHS-Funded tobacco dependence service intervention**

Are you aware of the effect the intervention is having on the service users?

- What feedback are you receiving? Examples?

Do you and your colleagues feel the intervention is worthwhile?

- Why do you feel that? Examples?

Can you tell me about the impact that this intervention has had on your work? What are the advantages/disadvantages?

What do you think can be done to improve the future of the NHS-funded tobacco dependency service intervention?

Do you feel there is anything that you and your colleagues can do to ensure the intervention is more effective? Anything that you would change?

**ANY FURTHER INFORMATION:**

Have you found any unintended consequences of implementing the NHS-funded tobacco dependency intervention? Either positive or negative

Have you discovered any other barriers or enables that have not been discussed already?

Anything else that you would like to add that has not already been discussed?

Thank you
