# Supplementary material for: Implementation and impact of NHS-funded tobacco dependence services in England: a mixed-method evaluation protocol
Source: BMJ Open. 2024 Dec 26;14(12):e089630. doi: 10.1136/bmjopen-2024-089630 (PMC11683999; doi:10.1136/bmjopen-2024-089630)
Supplement: online supplemental file 3 [file bmjopen-14-12-s003.docx]

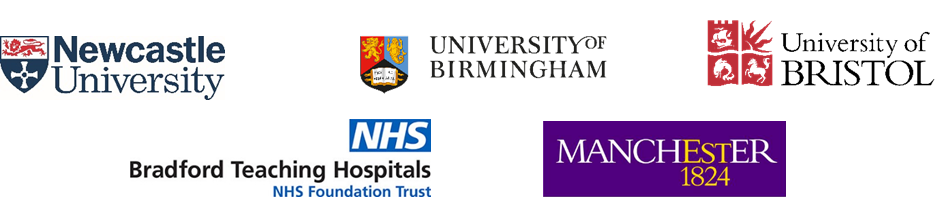


**Supporting the NHS Long Term Plan: An Evaluation of the Implementation and Impact of NHS-funded tobacco dependence services**

WPA3: Service users interview topic guide

Key: B – those not offered NHS-funded tobacco dependence service; C – those offered the service but decline; D – those offered the service and accepted

Thank you for agreeing to take part in this interview, and for giving up your time. We are inviting smokers who have recently been an inpatient in either an acute or mental health setting or have been in contact with maternity services, so I am pleased that we are meeting today. We are interested in finding out more about the stop smoking services you were offered or have previously accessed.

1. **History, Setting and Background (These questions are for all smokers)**

These first questions are going to be around you and the smoking services you have accessed before.

Are you currently a smoker? If so how long have you smoked? If you have recently quit, how long had you smoked?

Have you recently been an inpatient within an acute setting, mental health setting or recently accessed maternity services?

Where you asked if you smoked?

What help/support were you offered around smoking?

What support were you offered when you were discharged from [setting]?

Did this include any advice on stop smoking services in your area, their contact details etc?

Were you referred to the NHS-funded tobacco dependency service? (if **No**, ask questions in block **B**)

If **yes**, did you accept the service? (if **No**, ask questions in block **C**, if **yes**, ask questions in block **D**)

1. **QUESTIONS FOR THOSE WHO WERE NOT OFFERED THE NHS-FUNDED TOBACCO DEPENDENCE SERVICE**

What do you feel you need to help stop smoking?

Have you previously accessed any stop smoking services? If so, what services?

How did you access these services? How did you find out about them?

How did these services operate? What was provided to you?

What barriers did you find when accessing these services?

What barriers do you face when accessing stop smoking services now?

**Provide summary of the NHS-funded stop smoking service**

**Summary:** The NHS-funded tobacco dependence is a new stop smoking service that is based in acute inpatients, mental health inpatients and maternity settings. When a patient is admitted they are asked a series of questions to determine if they smoke. They are then referred to the on-site tobacco dependency advisors who carry out an assessment and aid the smoker in attempting to quit, through providing behavioural support and nicotine replacement therapy. When the patient is discharged into the community, a referral is made to the local stop smoking services who then assist the service user in the community to remain smoke free by providing further behavioural support and nicotine replacement therapy.

**Affective Attitude***: How an individual feels about the intervention*

Having explained the new NHS-funded tobacco dependence service, what are your thoughts about a service that would offer everything mentioned?

How would you feel about engaging with a service that operated like this service?

Can you tell me if you think this service could and would be successful in helping people to stop smoking?

What are your thoughts on the current stop smoking services and this new one, do you think there is a need for this new one?

**Burden:** *the perceived amount of effort required to participate in the intervention*

Thinking back to the new NHS-funded tobacco dependence service that I explained, how much effort do you think would be needed on the part of the smoker to engage with the service?

Thinking of the service explained, do you think the amount of effort that a smoker puts into the service will determine how much they get out of it?

**Ethicality**: *the extent to which the intervention has a good fit with an individuals value system*

What are your beliefs on the amount of effort and time that would be required to engage with the service?

If you would have been offered the service would you have accepted?

Can you explain why

Do you believe that this service has the potential to help people stop smoking?

Can you explain why?

Do you believe that this service would help you to stop smoking?

Can you explain why?

**Intervention Coherence:** *the extent to which the participant understands the intervention and how it works*

You are aware of the stop smoking services in your area, can you explain to me how you believe the new service fits in with the current services?

Ae you able to explain the differences between accessing the new service and the current stop smoking services in your area?

Do you believe you have reasonable knowledge of the stop smoking services in your area, and the new service?

**Opportunity costs:** *the extend to which benefits profits or values must be given up to engage in the intervention*

Can you tell me what you think you would have to give up to participate in a service like the one explained? (i.e., time, travel and friendships with smokers)

What ‘costs’ do you perceive would be associated with this service? (these do not need to be financial costs, but also emotional and social)

**Perceived effectiveness**: *the extent to which the intervention is perceived as likely to achieve its purpose*

Can you tell me how much you think the service would help you stop smoking?

What are your beliefs on the service helping other people to stop smoking?

What do you think could be done to make the intervention more useful for people who smoke?

**Self-efficacy*:*** *the participants confidence that they can perform the behaviour(s) required to participate in the intervention*

What skills, knowledge or level of confidence do you think is required to participate in the service?

Can you tell me do you think you have those skills, knowledge, and confidence?

Can you explain why?

Do you think other people who smoke posses the skills, knowledge, and confidence to engage with the service?

Can you explain why?

1. **QUESTIONS FOR THOSE WHO WERE OFFERED THE NHS-FUNDED TOBACCO DEPENDENCE SERVICE BUT DECLINED**

Can you tell me how was the intervention explained to you?

What influenced your decision to decline the intervention?

What could have been done differently for you to accept?

Have you previously accessed any stop smoking services? If so, what services?

How did you access these services? How did you find out about them?

How did these services operate? What was provided to you?

What barriers did you find when accessing these services?

What barriers do you face when accessing stop smoking services now?

**Affective Attitude***: How an individual feels about the intervention*

Are you able to tell me what you know about the service? (if this is limited, then please provide summary of service)

What are your thoughts about the service you declined?

Could anything be done differently for you to want to engage in this service?

Can you tell me if you think this service could and would be successful in helping people to stop smoking?

What are your thoughts on the current stop smoking services and this new one, do you think there is a need for this new one?

**Burden:** *the perceived amount of effort required to participate in the intervention*

Thinking back to the new service you declined, how much effort do you think would be needed on the part of the smoker to engage with the service?

Do you think the amount of effort that a person who smokes puts into this service will determine how much they get out of it?

**Ethicality**: *the extent to which the intervention has a good fit with an individuals value system*

What are your beliefs on the amount of effort and time that would be required to engage with the service?

Do you believe that this service has the potential to help people stop smoking?

Can you explain why?

Do you believe that this service would help you to stop smoking?

Can you explain why?

**Intervention Coherence:** *the extent to which the participant understands the intervention and how it works*

You are aware of the stop smoking services in your area, can you explain to me how you believe the new service fits in with the current services?

Ae you able to explain the differences between accessing the new service and the current stop smoking services in your area?

Do you believe you have reasonable knowledge of the stop smoking services in your area, and the new service?

**Opportunity costs:** *the extend to which benefits profits or values must be given up to engage in the intervention*

Can you tell me what you think you would have to give up to participate in this service? (i.e., time, travel and friendships with smokers)

What ‘costs’ do you perceive would be associated with this service? (these do not need to be financial costs, but also emotional and social)

**Perceived effectiveness**: *the extent to which the intervention is perceived as likely to achieve its purpose*

Can you tell me how much you think the service would help you stop smoking?

What are your beliefs on the service helping other people to stop smoking?

What do you think could be done to make the intervention more useful for people who smoke?

**Self-efficacy*:*** *the participants confidence that they can perform the behaviour(s) required to participate in the intervention*

What skills, knowledge or level of confidence do you think is required to participate in the service?

Can you tell me do you think you have those skills, knowledge, and confidence?

Can you explain why?

Do you think other people who smoke possess the skills, knowledge, and confidence to engage with the service?

Can you explain why?

1. **QUESTIONS FOR THOSE WHO WERE OFFERED THE NHS-FUNDED TOBACCO DEPENDENCE SERVICE AND ACCEPTED**

Can you tell me how was the intervention explained to you?

What influenced your decision to commit to the intervention?

Can you tell me how the service ran for you?

Is there anything that you would have rather been offered?

How long were you interacting with this service?

Can you tell me about the services you are currently accessing now (to support you in your quit attempt)?

**Affective Attitude***: How an individual feels about the intervention*

Can you tell me what you think/what your thoughts are on the service?

Can you tell me how you were feeling about starting the intervention?

Can you tell me how successful you found the service?

What are your thoughts in the need for this service to help people stop smoking?

**Burden**: *the perceived amount of effort required to participate in the intervention*

Can you explain to me how much effort you thought you would need to engage with the service?

Can you explain to me how much effort was required on your part to engage with the service?

Where these the same?

**Ethicality**: *the extent to which the intervention has a good fit with an individual’s value system*

Can you explain to me if you believe the service can work to help people stop smoking?

Do you think the service is worth the effort required?

Why do you think that?

**Intervention Coherence**: *the extent to which the participant understands the intervention and how it works*

Are you able to explain to me the service that was offered to you?

Can you explain to me what was required of you to actively participate?

Can you explain to me what was required of the healthcare staff?

Can you tell me how the service was explained to you?

How well do you know the service now?

**Opportunity costs**: *the extent to which benefits, profits or values must be given up to engage in the intervention*

Can you tell me what you had to give up to participate in the service effectively? (i.e., travel costs, time, friendships with smokers)

Can you tell me about the additional ‘costs’ – not just financial, but also social and emotional, that you had to ‘pay’ to participate in the service?

Do you think the costs acquired were worth the outcome of being smoke free?

**Perceived effectiveness:** *the extent to which the intervention is perceived as likely to achieve its purpose*

Do you feel the intervention will be successful in helping you stop smoking?

Can you tell me why you felt the intervention would be successful in aiding you to stop smoking?

Do you feel the intervention will be successful in helping other people stop smoking?

Can you tell me why you feel the intervention will/will not help other people stop smoking?

Can you tell me how did the intervention improve your knowledge of smoking, and aid in you stop smoking?

**Self-Efficacy**: *the participants’ confidence that they can perform the behaviour(s) required to participate in the intervention*

What skills and level of confidence do you think is needed to be successful in stop smoking?

Can you tell me if you believe you have those skills and confidence?

Why is that? Example?

Looking back, did you have any uncertainties around your ability to engage with the service?

Would you recommend the service to a family member or friend?

Were there any unexpected actions from this intervention that you have not expected?

Can you explain?
