# Supplementary material for: Implementation and impact of NHS-funded tobacco dependence services in England: a mixed-method evaluation protocol
Source: BMJ Open. 2024 Dec 26;14(12):e089630. doi: 10.1136/bmjopen-2024-089630 (PMC11683999; doi:10.1136/bmjopen-2024-089630)
Supplement: Supplementary file 4 [file bmjopen-2024-089630.reviewer_comments.pdf]

PEER REVIEW HISTORY

BMJ Open publishes all reviews undertaken for accepted manuscripts. Reviewers are asked to complete a checklist review form and are provided with free text boxes to elaborate on their assessment. These free text comments are reproduced below.

ARTICLE DETAILS

Title (Provisional)

Implementation and impact of NHS-funded tobacco dependence services in England: a mixed-method evaluation protocol

Authors

Aquino, Maria Raisa Jessica; Brennan-Tovey, Kerry; Fong, Mackenzie; Wearn, Angela; Bigirumurame, Theophile; Robinson, Tomos; Trevor, Miranda; Feeney, Joanna; Rutter, Ailsa; Sharrock, Ruth; West, Jane; Bridges, Sally; Attwood, Angela S; Jolly, Kate; Damery, Sarah; Flanagan, Sarah; Armitage, Chris; Russell, Samantha; Strong, Steve; Ramsay, Sheena E; Kaner, Eileen F.S

VERSION 1 - REVIEW

**Reviewer**	**1**
**Name**	**Togawa, Kayo**
**Affiliation**	**National Cancer Center Japan**
**Date**	**01-Jul-2024**
**COI**	**None**

Thank you very much for the opportunity to review the manuscript titled “Implementation and impact of NHS-funded tobacco dependence services in England: multi-method evaluation protocol.” This protocol is for an evaluation of the NHS-funded tobacco dependence services in England. The manuscript is well written but I have several questions which other readers might also ask. Below are my questions and comments to the authors for consideration.

- Figure 1 includes “smoking related illness and deaths,” etc. as part of the long-term outcome and impacts. I would like to know specific health outcomes that will be assessed (page 11) and how those outcomes will be assessed (e.g., medical record review, self-report).

- There are a number of outcomes that are not described in the text but are listed in Figure 1. It would be better to clarify whether all of them are part of the present study.

- Is there a target sample size for the quantitative part of the study?

- An illustration of study flow would be helpful.

- How will partners' smoking status be assessed?

- How were these specific settings were selected for evaluation? Were other settings considered, such as cancer screening and treatment?

- In Table 2, there is WP2 a, b, c – it would be easier for the readers if the same notions are used in the text.

Reviewer 2

Name Ravara, Sofia

Affiliation University of Beira Interior (UBI), Health Sciences Research Centre (CICS), Faculty of Health Sciences (FCS)

Date 19-Sep-2024

COI I have no conflict of interests

The manuscript describes a well-planned research protocol aiming to evaluate the implementation and impact of comprehensive tobacco dependence treatment in NHS acute settings (acute hospital, maternity, inpatient mental health) across five regions in England. This evaluation study is relevant and can potentially advance tobacco dependence treatment in England.

The study design and methodology are well described and adequate to the study aims; no major flaws exist. The manuscript reads rather well.

I suggest including a visual timeline for the research activities, and estimating the duration and effort of each work package tasks. The abstract should briefly include information about when the study should begin and end.

VERSION 1 - AUTHOR RESPONSE

Reviewer 1 comments:	Author response:
1. Thank you very much for the opportunity to review the manuscript titled "Implementation and impact of NHS-funded tobacco dependence services in England: multi-method evaluation protocol." This protocol is for an evaluation of the NHS-funded tobacco dependence services in England. The manuscript is well written but I have several questions which other readers might also ask.	Thank you for your feedback and clarifications.
2. Figure 1 includes "smoking related illness and deaths," etc. as part of the long-term outcome and impacts. I would like to know specific health	Thank you for your query. With respect to the outcome 'smoking related illness and deaths' on Fig. 1,

outcomes that will be assessed (page 11) and how those outcomes will be assessed (e.g., medical record review, self-report).	this is the anticipated outcome of the service as described in the NHS Long Term Plan. It is included in our logic model to illustrate the implementation of the programme, but these long-term outcomes are beyond the scope of our evaluation. We have revised the manuscript to reflect this (page 5):<br>The logic model presents the anticipated outcomes of implementing this intervention in the short, medium, and long-term; however not all outcomes will be assessed as part of this evaluation. Details on outcomes relevant to this evaluation are reported in their respective WPs (where applicable).<br><br>We will be looking at process and behavioural (i.e., quit) outcomes as outlined on page 12, lines 51-60 and page 13, lines 1-8 of the original submission. Individual patient level data will be collected from each participating NHS Trust (drawn from medical records). To clarify this to readers, we have edited the manuscript to reflect these points (pages 10-11 of revised manuscript):<br><br>Data will be collected across acute, maternity and mental health settings. These will include individual patient level data drawn from medical records.<br><br>[…]<br><br>Behaviour/health outcomes: Post-discharge smoking status. 28-day smoking status, carbon monoxide-validated smoking status, smoke-free births). In acute inpatient and mental health inpatient will include either 28-day smoking status, or self-reported quit. In maternity settings, quit data will include carbon monoxide-validated smoking status, smoking status at 36-weeks, smoke-free births, or self-reported quit.

	[…]<br><br>Data on quit rates will be collected across acute, maternity and mental health settings. Quit data for acute inpatient and mental health inpatient will include either 28-day smoking status, or self-reported quit. In maternity settings, quit data will include carbon monoxide-validated smoking status, smoking status at 36-weeks, smoke-free births, or self-reported quit. All individual patient level data will be drawn from medical records.
3. There are a number of outcomes that are not described in the text but are listed in Figure 1. It would be better to clarify whether all of them are part of the present study.	Thank you for this comment. As in the preceding point, these are the anticipated outcome of the service as described in the NHS Long Term Plan. It is included in our logic model to illustrate the implementation of the programme, but these long-term outcomes are beyond the scope of our evaluation. We have revised the manuscript to reflect this, **please see response to point 2**.
4. Is there a target sample size for the quantitative part of the study?	Thank you for this clarification. No formal sample size calculation was conducted. However, given the number of patients admitted to 11 NHS Trusts (i.e., the expected number of Trusts to participate in this evaluation, n= 977,500), up to 7 months of submissions (at the time of estimation) and a smoking rate of 12.1% in England, there are approximately 69,000 potential smokers for inclusion in the evaluation.<br><br>We have included this information in the manuscript (pages 10-11): While no formal sample size calculation was conducted, we estimate approximately 69,000 potential individuals for inclusion in the evaluation. This estimate was based on 11 participating NHS Trusts, approximately 7 months of data collection (taking into consideration

	potential data governance and submission delays/gaps), and a national smoking prevalence of 12.1% in England.
5. An illustration of study flow would be helpful.	Thank you for your suggestion. We have now included a flowchart on page 12 of the revised manuscript.
6. How will partners' smoking status be assessed?	While the offer is also open to pregnant women's/individuals' partners; the particulars of how smoking status is assessed is outside the scope of the evaluation. The Saving Babies Lives Care Bundle states: *If her partner or other household members smoke, they can support her by making efforts to give up smoking too and have a smoke-free home. Find out about local stop smoking support available for women and families in your area and adopt an opt-out approach to smoking cessation* (NHS England » Saving babies' lives: version 3)
7. How were these specific settings were selected for evaluation? Were other settings considered, such as cancer screening and treatment?	Thank you for this query. The settings selected are all the settings in which the NHS Long Term Plan is being implemented. The focus of this evaluation is the implementation of the NHS Long Term Plan across all these settings.
8. In Table 2, there is WP2 a, b, c – it would be easier for the readers if the same notions are used in the text.	Thank you. We have updated the headings within the text to reflect labels used in Table 2 (page 9 of revised manuscript):<br><br>*WP2a - Survey*<br>*WP2b – Interviews with healthcare professionals*<br>*WP2c – Interviews with smokers*
**Reviewer 2 comments:**	**Author response:**
1. The manuscript describes a well-planned research protocol aiming to evaluate the implementation and impact of comprehensive tobacco dependence treatment in NHS acute settings (acute hospital, maternity, inpatient mental health) across five regions in England. This evaluation study is relevant and can potentially advance tobacco dependence treatment in England.<br>The study design and methodology are well described and adequate to the study aims; no major flaws exist. The manuscript reads rather well.	Thank you for your kind words and feedback.

2.	I suggest including a visual timeline for the research activities, and estimating the duration and effort of each work package tasks.	Thank you. We provided a study flowchart (Fig. 2) with visual projected timeline on page 12 of the revised manuscript. We have also added information on the duration of programme set up activities and described the indicative duration of each WP within the main text:<br>Programme set up – pre-evaluation activities *(Months 1-12)*<br>The first year of funding will be used to appoint researchers across the evaluation regions, obtain the relevant ethical approvals and data sharing agreements, and complete procedures for setting up participating NHS Trusts.<br><br>WP 1: Key informant interviews and document analysis *(Months 13-24)*<br><br>WP 2: Surveys and interviews with healthcare professionals and interviews with smoking individuals *(Months 25-48)*<br><br>WP 3: Effectiveness and process analysis *(Months 25-48)*<br><br>WP 4: Health economic analysis *(Months 25-48)*
3.	The abstract should briefly include information about when the study should begin and end.	Thank you for this. We have now included the study period in the abstract (page 1 of revised manuscript):<br>It is funded from September 2021-September 2025.
